# Studies of Dynamic Protein-Protein Interactions in Bacteria Using *Renilla* Luciferase Complementation Are Undermined by Nonspecific Enzyme Inhibition

**DOI:** 10.1371/journal.pone.0043175

**Published:** 2012-08-15

**Authors:** Stavroula K. Hatzios, Simon Ringgaard, Brigid M. Davis, Matthew K. Waldor

**Affiliations:** 1 Division of Infectious Diseases, Brigham and Women’s Hospital, Boston, Massachusetts, United States of America; 2 Department of Microbiology and Immunobiology, Harvard Medical School, Boston, Massachusetts, United States of America; 3 Howard Hughes Medical Institute, Boston, Massachusetts, United States of America; University of Illinois at Chicago College of Medicine, United States of America

## Abstract

The luciferase protein fragment complementation assay is a powerful tool for studying protein-protein interactions. Two inactive fragments of luciferase are genetically fused to interacting proteins, and when these two proteins interact, the luciferase fragments can reversibly associate and reconstitute enzyme activity. Though this technology has been used extensively in live eukaryotic cells, split luciferase complementation has not yet been applied to studies of dynamic protein-protein interactions in live bacteria. As proof of concept and to develop a new tool for studies of bacterial chemotaxis, fragments of *Renilla* luciferase (Rluc) were fused to the chemotaxis-associated response regulator CheY3 and its phosphatase CheZ in the enteric pathogen *Vibrio cholerae*. Luciferase activity was dependent on the presence of both CheY3 and CheZ fusion proteins, demonstrating the specificity of the assay. Furthermore, enzyme activity was markedly reduced in *V. cholerae* chemotaxis mutants, suggesting that this approach can measure defects in chemotactic signaling. However, attempts to measure changes in dynamic CheY3-CheZ interactions in response to various chemoeffectors were undermined by nonspecific inhibition of the full-length luciferase. These observations reveal an unexpected limitation of split Rluc complementation that may have implications for existing data and highlight the need for great caution when evaluating small molecule effects on dynamic protein-protein interactions using the split luciferase technology.

## Introduction

Genetically encoded reporters have revolutionized the study of bacterial protein-protein interactions. Two-hybrid systems, which couple transcriptional activation of a reporter gene to protein binding, have been widely used to characterize novel interaction partners [1]. Protein fragment complementation assays (PCA), in which two inactive fragments of a monomeric reporter, such as β-galactosidase or green fluorescent protein, are fused to interacting proteins, have also been used to measure binding interactions [2], [3]. However, both of these approaches typically suffer from limitations (e.g., lag time of transcriptional activation or fluorescent protein maturation, irreversible signal amplification over time) that preclude real-time analyses of dynamic interactions. Fluorescence and bioluminescence resonance energy transfer (FRET and BRET, respectively) assays can overcome these limitations [4], [5], but are more technically demanding. Thus, a tractable assay that enables quantitative and sensitive measurements of dynamic interactions in real-time would be a powerful addition to the existing toolkit for studies of bacterial protein interactions.

Luciferase protein fragment complementation (a.k.a. split luciferase complementation) is a recently described PCA that has been used to analyze dynamic protein-protein interactions in live mammalian cells [6]–[10]. This assay relies on the division of a bioluminescent luciferase enzyme into two inactive fragments that are genetically fused to interacting proteins. When these two proteins interact, the luciferase fragments can associate, thereby reconstituting luciferase activity. Importantly, unlike fluorescent protein complementation strategies, split luciferase complementation is reversible; it can be used to monitor protein-protein interactions in real-time, unlike two-hybrid approaches and most other protein fragment complementation assays; finally, unlike FRET/BRET, it does not require monitoring of filtered light emissions [3], [10]–[12]. Notably, this technology was recently adapted to monitor the assembly of a stable protein complex in *Salmonella* [13].

We hypothesized that split luciferase complementation could represent a versatile approach to studying dynamic protein-protein interactions in bacteria. Though several luciferase enzymes have been developed into split reporters, we chose to use recently described fragments of *Renilla reniformis* luciferase (Rluc) [11] for our assay because the light-emitting substrate of Rluc, coelenterazine (clz), can easily pass through the cell envelope of Gram-negative bacteria [13], [14], unlike the more common luciferase substrate D-luciferin, which requires acidic pH for optimal membrane permeability [15], [16]. As a first application for proof of concept, we fused these fragments to two chemotaxis proteins from *Vibrio cholerae,* the curved Gram-negative bacterium that causes human cholera.

Classically, bacterial chemotaxis reflects the sensing of external stimuli by membrane-associated receptors that transmit a signal through a cytoplasmic cascade that modulates flagellar rotation [17]. Membrane-embedded methyl-accepting chemotaxis proteins (MCPs) initiate chemotactic signaling upon detecting a change in local chemical gradients. Binding of a chemorepellent (or a decrease in chemoattractant binding) to its cognate MCP induces a conformational change that leads to autophosphorylation of a cytosolic kinase, CheA. CheA subsequently phosphorylates the response regulator CheY, which diffuses across the cell and binds the flagellar motor switch protein FliM. This binding switches the direction of flagellar rotation, which induces random reorientation of the cell. The lifetime of phosphorylated CheY is tightly regulated by the phosphatase CheZ, which hydrolyzes CheY’s phosphate group, thereby terminating the chemotactic signal. Pathway activity is also regulated by methyltransferase and methylesterase proteins (CheR and CheB, respectively), which modulate pathway sensitivity. Collectively, these proteins allow bacteria to maintain their direction (straight swimming) under favorable conditions, and alter their direction (tumbling) under adverse conditions.

*V. cholerae* encodes an unusually large number of putative MCPs, as well as three potentially independent clusters of downstream signaling proteins (chemotaxis clusters I, II, and III) [18], [19]. Several cluster II genes, such as *cheY3* and *cheA2*, are required for chemotaxis in vitro [20]; however, genes from the other two clusters remain largely uncharacterized, and ligands have not been identified for any of the receptors.

**Figure 1:**
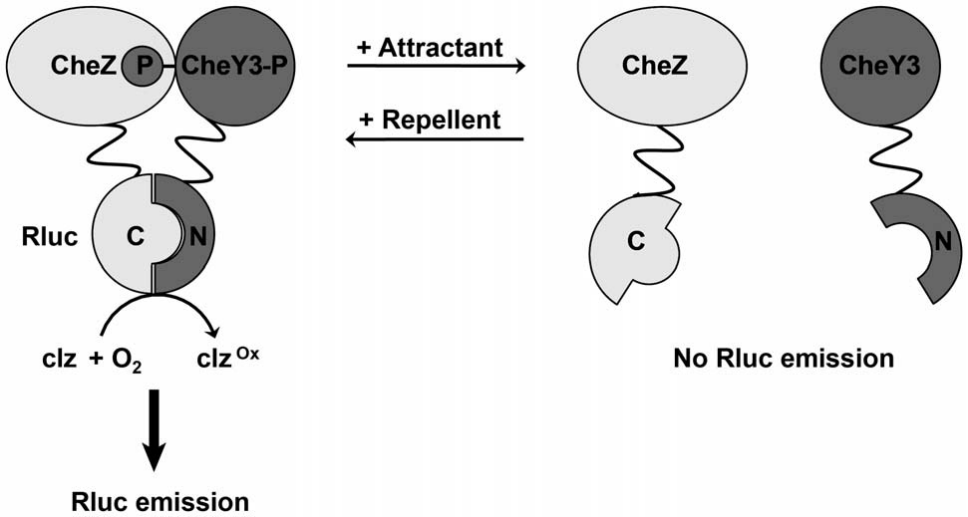
Design of an Rluc-based PCA for studies of *Vibrio cholerae* chemotaxis. CheY3 and CheZ proteins fused to inactive Rluc N- and C-terminal fragments, respectively, associate upon CheY3 phosphorylation, reconstituting full-length Rluc, which oxidizes the light-emitting substrate coelenterazine (clz).

Progress in studies of bacterial chemotaxis has been hindered by a dependence on low-throughput and/or semi-quantitative chemotaxis assays (i.e., capillary assays, soft-agar swarm plate assays) [21], [22]. An assay based on the split luciferase technology could provide a sensitive, quantitative, and rapid microplate-based approach to studying bacterial chemotaxis, with particular utility for characterizing novel chemoeffectors. Importantly, elegant BRET and FRET analyses of chemotaxis by Berg and colleagues have demonstrated that the interaction of CheY and CheZ proteins in *E. coli* is directly proportional to chemotaxis pathway activity [14], [23]. However, due to their relative technical complexity, these assays are not widely used or easily adapted to a high-throughput format. We hypothesized that fusion of Rluc fragments to homologous proteins from *V. cholerae* would enable a direct measure of chemotactic responses and provide a more tractable platform for chemoeffector characterization (Figure 1).

Here, we demonstrate that an Rluc-based PCA can be used to measure CheY3-CheZ interactions in *V. cholerae* and that this approach can quantify differences in chemotactic signaling. However, nonspecific inhibition of Rluc activity by small molecule effectors compromises the utility of this technique in measuring dynamic protein-protein interactions. These findings uncover a critical limitation of split luciferase complementation that may have broad implications for existing and future applications of this technology.

## Materials and Methods

### Growth Conditions and Media

*V. cholerae* and *E. coli* were grown at 37°C in LB medium or on LB agar plates supplemented as needed with the following antibiotics: 5 µg/mL chloramphenicol and 200 µg/mL streptomycin (*V. cholerae*); 20 µg/mL chloramphenicol (*E. coli*).

### Strains and Plasmids

A complete list of the strains, plasmids, and primers used in this study can be found in the Supplemental Information (Table S1, S2, and S3). The *V. cholerae* El Tor clinical isolate C6706 was used to generate all *V. cholerae* mutant strains. The *E. coli* strains DH5αλ*pir* and SM10λ*pir* were used for cloning and for conjugation, respectively, and MG1655 was used for bioluminescence assays.

Strain SR28 (Δ*che2*, a deletion which encompasses the entire *V. cholerae* chemotaxis cluster II) was generated using standard allele exchange techniques as previously described [24]. Strains SR33 (Δ*che1che2che3*) and Δ*cheY3cheZ* were similarly prepared using derivatives of plasmid pCVD442 [25]. Strain SR33 was created by consecutive deletions of chemotaxis clusters I, II, and III using plasmids pSR1157, pSR1020, and pSR1158, respectively. Strain Δ*cheY3cheZ* was created by simultaneously deleting the adjacent *cheY3* and *cheZ* genes of chemotaxis cluster II using plasmid pCVD442-Δ*cheY3cheZ*. Plasmids pSR1157, pSR1020, pSR1158, and pCVD442-Δ*cheY3cheZ* were conjugated into *V. cholerae* using *E. coli* strain SM10λ*pir* [26].

Plasmid pSR1157 was constructed by PCR amplification of the up- and downstream regions flanking genes *vc1394* and *vc1406*, respectively, using primer pairs che1-cc/che1-dd and che1-aa/che1-bb and *V. cholerae* C6706 genomic DNA as template. Equal amounts of these PCR products were combined and used as template in a third PCR reaction using primers che1-aa and che1-dd. The resulting product was digested with XbaI and ligated into similarly digested pCVD442 plasmid to generate pSR1157. Plasmid pSR1020 was constructed as previously described [24]. Plasmid pSR1158 was constructed by PCR amplification of the up- and downstream regions flanking genes *vca1088* and *vca1096*, respectively, using primer pairs che3-cc/che3-dd and che3-aa/che3-bb and *V. cholerae* C6706 genomic DNA as template. Equal amounts of these PCR products were combined and used as template in a third PCR reaction using primers che3-aa and che3-dd. The resulting product was digested with XbaI and ligated into similarly digested pCVD442 plasmid to generate pSR1158.

Plasmid pCVD442-Δ*cheY3cheZ* was constructed by PCR amplification of the regions flanking the *cheY3* (*vc2065*) and *cheZ* (*vc2064*) genes of chemotaxis cluster II using *V. cholerae* C6706 genomic DNA as template. Primers SKH-51 and SKH-52 were used to amplify the region downstream of *cheY3*, and primers SKH-53 and SKH-54 were used to amplify the region upstream of *cheZ*. Equal amounts of these PCR products were then combined and used as template in a final PCR reaction with primers SKH-51 and SKH-54. The resulting product was digested with XbaI and ligated into similarly digested pCVD442 plasmid to generate pCVD442-Δ*cheY3cheZ*.

The *rluc* gene was cloned from the pRL-null vector (Promega). The *cheY3* and *cheZ* genes were cloned from *V. cholerae* C6706 genomic DNA. Split Rluc complementation fragments RlucN and RlucC corresponded to amino acids 1–110 and 111–311, respectively. A T2A mutation was introduced in RlucN to preserve the amino acid sequence used in the original split Rluc construct [11], [27]. Fusions to CheY3 and CheZ were constructed by PCR using primers with complementary overhangs. Following initial amplification of each chemotaxis gene and Rluc fragment, a second round of PCR was used to anneal *cheY3* with *rlucN* and *cheZ* with *rlucC*. A ribosome-binding site was introduced upstream of *cheZ* (and the *rlucC* fragment of the control plasmid) to enable tandem assembly of both Rluc fusions (and the Rluc fragments alone) into a single expression vector. The *cheY3-rlucN* and *cheZ-rlucC* inserts were sequentially introduced into pBAD33 (chloramphenicol resistant) under an arabinose-inducible promoter using the KpnI/XbaI and XbaI/SphI restriction sites, respectively, to give plasmid pYNZC. The *rlucN* and *rlucC* inserts were cloned into pBAD33 using the same restriction sites to give plasmid pRlucN-RlucC. Plasmid pRluc was constructed by PCR amplification of the *rluc* gene using primers SKH-71 and SKH-76 followed by digestion with KpnI/SphI and ligation into similarly digested pBAD33. The final constructs were confirmed by DNA sequencing and transformed into *V. cholerae* and *E. coli* via electroporation.

### Preparation of Cells for Bioluminescence Assays

An overnight culture of *V. cholerae* or *E. coli* was prepared in 2-mL LB supplemented with chloramphenicol and inoculated with cells from a frozen glycerol stock. The culture was incubated at 37°C with shaking at 250–275 rpm. The following day, the overnight culture was diluted 1∶100 in 5 mL fresh LB-chloramphenicol containing 0.2% (v/v) L-arabinose, which conferred optimal restoration of swarming activity in Δ*cheY3cheZ V. cholerae* cells, and grown for 3 h at 37°C on a rotary shaker to an OD∼1. The culture was washed twice with phosphate buffered saline (PBS; pH 7), adjusted to an OD∼0.4–0.5, and aliquoted into a white 96-well plate in triplicate.

### Bioluminescence Assays

Aliquots of native coelenterazine (clz) or benzyl-coelenterazine (clz-h) (NanoLight) dissolved in ethanol were stored at −80°C. Solutions (30 mM in distilled water) of D-glucose (American Bioanalytical), L-serine, L-alanine, L-arginine, D-mannose, and nickel chloride (Sigma) were prepared and used at a concentration of 1 mM. Prior to each assay, a single aliquot of clz/clz-h was thawed, diluted in PBS to a final concentration of 250 µM, and incubated in the dark at room temperature for 1 h to stabilize the clz/clz-h signal. To measure changes in split Rluc complementation resulting from small molecule-induced effects on CheY3-CheZ binding, we established conditions that would provide a stable split Rluc signal. Because the kinetics of Rluc luminescence are characterized by a rapid drop in signal intensity over time [14], obtaining a stable baseline is essential for comparing pre- and post-stimulation values. We determined that incubating cells with clz for 30 min at room temperature prior to initiating the bioluminescence read produced a consistently stable split Rluc signal. Thus, clz/clz-h was added to a final concentration of 7.5 µM to the PBS-washed cells, which were subsequently incubated in the dark for 30 min at room temperature to allow the luciferase-generated signal to stabilize. The total luminescence of each well was then measured every minute for a total of 5 min with an integration time of 1 sec using a SpectraMax L Luminescence Microplate Reader (Molecular Devices). Immediately thereafter, a single chemoeffector (or an equal volume of distilled water) was manually injected into each well and a second luminescence read was initiated as before. Data analysis was performed using Microsoft Excel.

### Western Blot Analysis

For analysis of CheY3-RlucN/CheZ-RlucC expression levels in different genetic backgrounds, 1 mL of cells from cultures of equal OD was centrifuged for 1 min at 15 krpm and frozen at −80°C. The following day, cells were resuspended in 150 µL 50 mM Tris buffer (pH 8) containing 1% (v/v) sodium dodecyl sulfate and incubated at 95°C for 5 min. Cells were lysed by sonication (10 1-sec pulses at an output setting of 5; Fisher Scientific Model 60 Sonic Dismembrator), treated with 4X NuPAGE LDS sample buffer (Life Technologies) containing 4 mM dithiothreitol, and incubated at 65°C for 10 min prior to SDS-PAGE and immunoblotting with the anti-Rluc antibody (1∶100,000 dilution; mouse monoclonal, clone 5B11.2; Millipore). Protein bands were detected using a horseradish peroxidase-conjugated secondary antibody (1∶2000 dilution; goat anti-mouse IgG-HRP, sc-2005; Santa Cruz Biotechnology) and chemiluminescent reagents (Pierce).

## Results

### Split Rluc Complementation Measures CheY3-CheZ Interaction in Vibrio cholerae

To explore the utility of split Rluc complementation for probing dynamic protein-protein interactions in *V. cholerae*, we applied the technology toward the goal of developing a novel chemotaxis assay that could in principle enable rapid, quantitative measurements of chemotaxis in response to chemotactic stimuli. Since previous BRET- and FRET-based analyses of chemotactic signaling in *E. coli* [14], [23] demonstrated that the interaction of the CheY and CheZ proteins is directly proportional to chemotaxis pathway activity, we reasoned that fusion of Rluc fragments to homologous proteins from *V. cholerae* (CheY3 and CheZ, respectively) would enable a direct measure of chemotactic responses. Notably, unlike existing chemotaxis assays, we envisioned that split Rluc complementation performed in a microplate-based format might also provide a tractable platform for high-throughput studies of *V. cholerae* chemotaxis.

**Figure 2:**
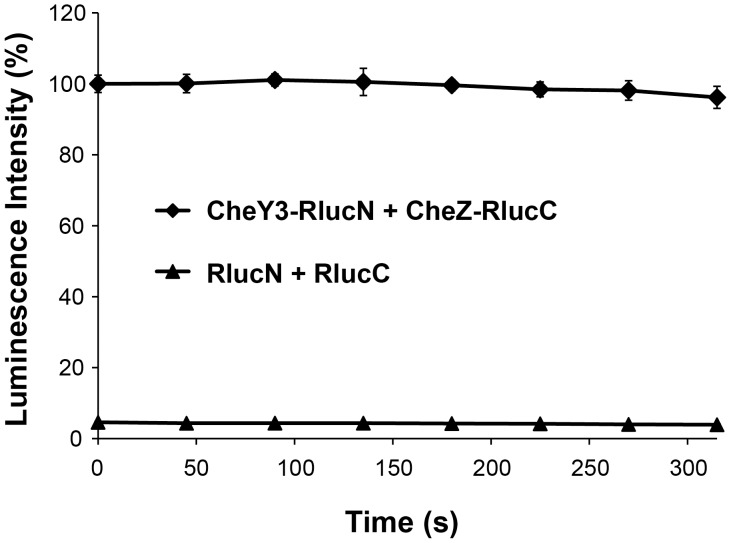
Split Rluc complementation is dependent on fusing Rluc N- and C-terminal fragments to CheY3 and CheZ. Luminescence generated by wild-type *V. cholerae* co-expressing CheY3-RlucN and CheZ-RlucC or unfused RlucN and RlucC. Data are reported as a percentage of the highest initial signal intensity and represent the average of three technical replicates.

For the design of our Rluc-based PCA, we used recently described Rluc fragments that provided sensitive, quantitative measurements of dynamic protein interactions with a high signal-to-noise ratio in mammalian cells [11]. Plasmids were constructed encoding the *V. cholerae* chemotaxis cluster II genes *cheY3* and *cheZ* fused to the N- and C-terminal fragments of Rluc (*rlucN* and *rlucC*), respectively, by a 14-amino acid flexible linker. RlucN was fused to either the N- or C-terminus of CheY3, while RlucC was fused to the C-terminus of CheZ; structural comparisons to homologous proteins from *E. coli* suggested this fusion strategy would yield the best split Rluc complementation upon binding of CheY3 to CheZ. These constructs, as well as various control constructs, were introduced into wild-type and Δ*cheY3cheZ V. cholerae* and evaluated for their ability to reconstitute luciferase activity.

The luciferase activity of cells co-expressing CheY3-RlucN and CheZ-RlucC far exceeded that generated by cells co-expressing unfused RlucN and RlucC (∼20-fold difference in luminescence intensity; Figure 2), suggesting that split Rluc complementation is dependent on CheY3-CheZ interaction. Expression of individual CheY3-RlucN or CheZ-RlucC fusion proteins produced negligible luciferase activity (data not shown). Cells expressing the RlucN-CheY3/CheZ-RlucC fusion pair grew poorly and were not analyzed further. Thus, the CheY3-RlucN/CheZ-RlucC construct, pYNZC, was used for all subsequent experiments. To further maximize reporter activity, expression of fusion proteins was induced with 0.2% arabinose, and the Rluc substrate that yielded the highest luminescence (i.e., clz) was selected (Figure S1). Notably, co-expression of CheY3-RlucN and CheZ-RlucC in wild-type *V. cholerae* generated luciferase activity comparable to that observed in the Δ*cheY3cheZ* mutant, demonstrating that the endogenous CheY3 and CheZ proteins do not significantly inhibit split Rluc complementation.

### CheY3-CheZ Interaction is Markedly Reduced in V. cholerae Chemotaxis Mutants

**Figure 3:**
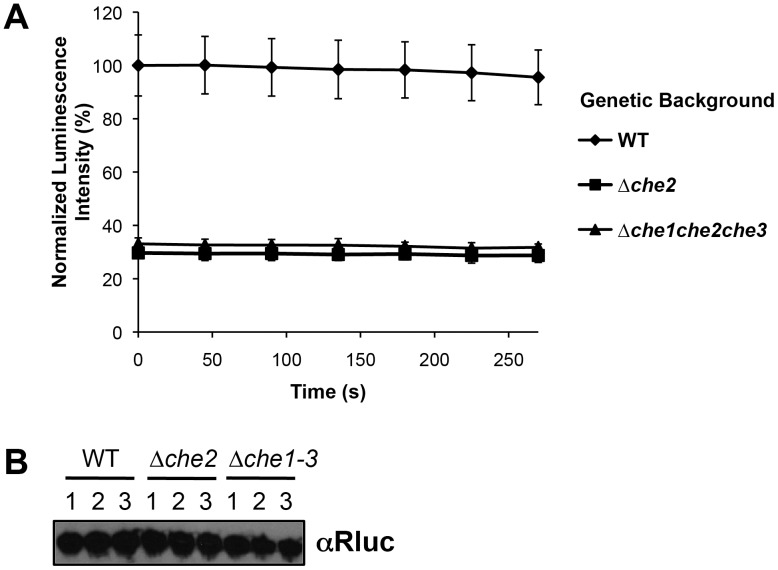
Rluc PCA detects reduced CheY3-CheZ interaction in *V. cholerae* chemotaxis mutants. (*A*) Luminescence generated by wild-type (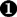),Δ*che2* (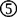), and Δ*che1che2che3* (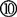) *V. cholerae* co-expressing CheY3-RlucN and CheZ-RlucC. Data from three biological replicates were normalized by optical density, averaged, and reported as a percentage of the highest initial signal intensity. (*B*) Western blot analysis of CheY3-RlucN/CheZ-RlucC expression in samples from (*A*). Only the CheZ-RlucC fusion protein was detected by the monoclonal anti-Rluc antibody.

We hypothesized that pYNZC could be used to measure changes in CheY3-CheZ interaction, and thus chemotactic signaling, in *V. cholerae* mutants compromised for chemotaxis. We introduced pYNZC into Δ*che2*, a strain lacking all cluster II chemotaxis genes (including *cheY3* and *cheZ*), and Δ*che1che2che3*, which lacks all three chemotaxis clusters. Because cluster II genes are required for chemotaxis under laboratory conditions [20], [24], both of these strains are non-chemotactic and were thus predicted to have significantly fewer CheY3-CheZ interactions than wild-type *V. cholerae*. As expected, the luciferase activities of both Δ*che2* and Δ*che1che2che3* were markedly lower than wild-type cells (∼70% reduction in luminescence intensity; Figure 3A). Immunoblotting confirmed that these differences were not due to variable expression of the luciferase fragments from pYNZC in these different genetic backgrounds (Figure 3B). Since Δ*che2* and Δ*che1che2che3* exhibited approximately equal luminescence intensities (Figure 3A), the additional chemotaxis signaling proteins encoded by cluster II (e.g., *cheA2*, *cheW1*) are presumably the primary mediators of CheY3-CheZ binding under these conditions. However, a low level of split Rluc complementation was still observed in these strains, raising the possibility that some interactions between CheY3 and CheZ can occur despite the absence of chemotaxis proteins that ostensibly regulate CheY3 phosphorylation (e.g., CheA2). Indeed, other factors, such as CheY acetylation [28], have been shown to affect chemotactic signaling in *E. coli* and may stimulate CheY3-CheZ binding in *V. cholerae* independent of other chemotaxis machinery. Still, the difference in reporter activity between wild-type and non-chemotactic strains appears to validate use of split Rluc complementation as an indicator of chemotactic signaling in different *V. cholerae* genetic backgrounds.

### Nonspecific Inhibition of Rluc Precludes Analysis of Dynamic CheY3-CheZ Interactions

**Figure 4:**
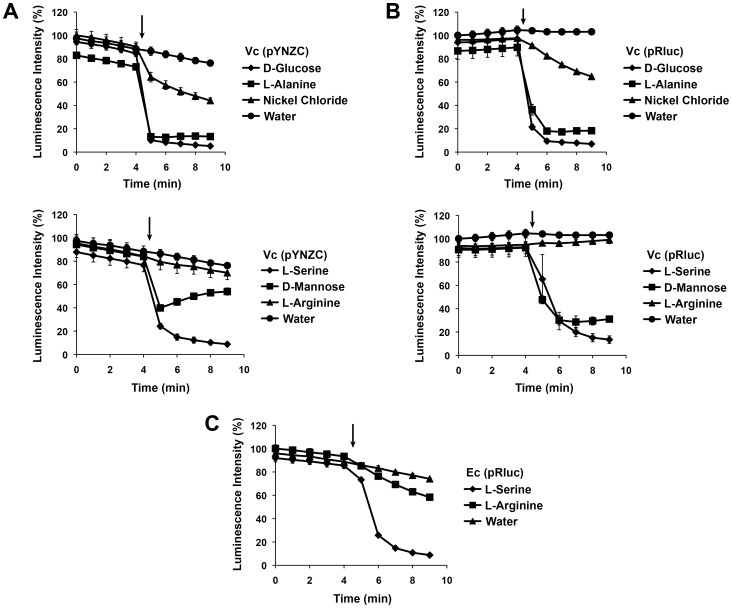
Apparent chemoattractant response of Rluc PCA reflects nonspecific inhibition of full-length Rluc. Luminescence generated by wild-type *V. cholerae* co-expressing CheY3-RlucN and CheZ-RlucC from pYNZC (*A*) or unfused, full-length Rluc from pRluc (*B*) was measured before and after treatment with 1 mM D-glucose, L-alanine, or nickel chloride (top panel) and 1 mM L-serine, D-mannose, or L-arginine (bottom panel) or an equal volume of water. Luminescence generated by wild-type *E. coli* expressing full-length Rluc from pRluc was measured before and after treatment with L-serine, L-arginine, or water (*C*). Reagent addition is denoted by an arrow. Data are reported as a percentage of the highest initial signal intensity and represent the average of three technical replicates.

To assess the utility of split Rluc complementation for monitoring changes in protein-protein interactions in *V. cholerae*, we investigated the effects of chemoeffector addition on pYNZC reporter activity. Based on the BRET- and FRET-based assays for studying CheY-CheZ interactions in *E. coli* [14], [23], we expected the luciferase activity generated by cells co-expressing CheY3-RlucN and CheZ-RlucC to be proportional to chemotaxis pathway activity and to reflect the reversible association of CheY3 and CheZ proteins. Thus, applying a chemoattractant to those cells should decrease CheY3 phosphorylation and the extent of CheY3-CheZ binding, resulting in a rapid loss of luminescence. To test this, we treated wild-type *V. cholerae* expressing these fusion proteins from pYNZC with several well-characterized chemoattractants, including L-serine and D-glucose [29]. These molecules stimulated a rapid (within one minute) and significant decrease in cellular luminescence relative to a water-only control (Figure 4A). Maximal effects were observed following addition of these factors at 1 mM; however, addition of known attractants at much lower concentrations (∼1 µM) also reduced luminescence (data not shown). Thus, chemoattractant addition appeared to reduce the interaction between CheY3 (presumably as CheY3-P) and CheZ as expected, suggesting that split Rluc complementation was indeed reversible.

Though initially promising, several aspects of the CheY3-CheZ response to chemoattractant addition were inconsistent with published reports of bacterial chemotactic signaling and prompted us to question the significance of these data. First, not all chemoeffectors had the anticipated effect on split Rluc complementation. For example, L-arginine, a strong *V. cholerae* chemoattractant [29], [30], did not decrease luciferase activity (Figure 4A). Furthermore, some common bacterial repellents, such as nickel chloride, cobalt chloride, and butyric acid [31], [32], which were expected to stimulate CheY3 phosphorylation, enhance binding to CheZ, and thus increase split Rluc complementation, instead elicited an “attractant-like” response (e.g., Figure 4A, nickel chloride). Finally, the signal recovery typically observed shortly after attractant addition in BRET- and FRET-based assays [14], [23], which reflects MCP adaptation to local chemical gradients, was notably absent from our split Rluc data. In light of these issues, we began to suspect that our Rluc PCA was not accurately reporting changes in CheY3-CheZ interactions in response to chemotactic stimuli.

To explore the possibility that certain chemoeffectors might nonspecifically inhibit Rluc activity, generating a false “attractant-like” response, we monitored the effect of chemoeffector treatment upon cells expressing the full-length enzyme. Remarkably, we observed a decrease in luminescence that mirrored nearly exactly the response of cells co-expressing CheY3-RlucN and CheZ-RlucC (compare Figure 4A and Figure 4B), thereby confirming our suspicion that nonspecific inhibition of Rluc was compromising the significance of the split Rluc data. Notably, we observed a similar inhibition profile in *E. coli* expressing the full-length luciferase (Figure 4C), indicating that this phenomenon is manifest in other bacteria. Furthermore, nonspecific inhibition of Rluc activity was observed regardless of the order of reagent addition. For example, pretreatment of cells expressing CheY3-RlucN/CheZ-RlucC or the full-length enzyme with L-serine followed immediately by clz addition and measurement of Rluc activity decreased luminescence intensity by at least 65% relative to cells pretreated with water or L-arginine. Thus, we were unable to validate the reversibility of CheY3-CheZ-mediated split Rluc complementation, precluding its utility as a tool for evaluating dynamic chemotactic responses.

## Discussion

We sought to develop a new method for quantitatively probing real-time protein-protein interactions in live bacteria using split luciferase complementation, a technique that has been widely applied to study protein-protein interactions in mammalian cells [3]. As proof of concept, we chose to develop an Rluc-based PCA for *V. cholerae* chemotaxis by fusing two inactive Rluc fragments to the chemotaxis response regulator CheY3 and its cognate phosphatase CheZ. In *E. coli*, BRET and FRET have been used to study the transient, phosphorylation-dependent interaction of CheY and CheZ proteins, which is directly proportional to chemotaxis pathway activity [14], [23]. We hypothesized that split Rluc complementation could provide an alternative means of studying these interactions in *V. cholerae*, establishing a rapid, highly tractable, microplate-based assay for bacterial chemotaxis with the potential for high-throughput applications.

Consistent with a previous study that used *Gaussia princeps* luciferase complementation to detect assembly of a stable protein complex in *Salmonella* [13], we found that fusion of Rluc fragments to known chemotaxis interaction partners enabled reconstitution of an active luciferase in *V. cholerae* cells (Figure 2). Furthermore, we discovered that the CheY3-CheZ-dependent signal was reduced in chemotaxis-deficient *V. cholerae* strains, suggesting that this system serves as a reporter of basal chemotactic signaling (Figure 3). However, our attempts to measure changes in chemotactic signaling in response to the addition of known chemoattractants, which should decrease CheY3-CheZ interactions, and hence, the associated split Rluc signal, were compromised by the unexpectedly broad small molecule inhibition profile of the full-length Rluc enzyme (Figure 4). These findings demonstrate the need for great caution in interpreting chemical effects on dynamic protein-protein interactions using split Rluc complementation.

Importantly, we performed our assays under conditions that yield a relatively stable split Rluc signal in order to more easily compare enzyme activity before and after chemoeffector addition. We determined that pre-incubation of cells with clz for 30 minutes prior to measuring luminescence was typically sufficient to achieve a stable signal (e.g., Figure 2 and Figure 3A), though a gradual decrease in Rluc activity was sometimes still observed despite this incubation period (e.g., Figure 4A). In mammalian cell-based Rluc PCAs, bioluminescence is typically measured immediately following clz addition because maximal luminescence occurs after an initial burst in Rluc activity (typically within the first minute of clz addition) [10], [11]. Though we obtained similar results using this approach (i.e., pretreatment of cells with L-serine followed by clz addition significantly decreased luminescence intensity relative to cells pretreated with water or L-arginine), we found that pre-incubation of the cells with clz facilitated more quantitative comparisons between different chemoeffectors. However, nonspecific inhibition of full-length Rluc was evident regardless of the order of reagent addition.

Chemical inhibition of luciferases, which has been previously documented, can complicate their use as both transcriptional and biochemical reporters [33]–[36]. Even so, we were greatly surprised by the breadth of the inhibition we observed, as the majority of the compounds we tested appeared to reduce Rluc activity. Notably, these inhibitory compounds (i.e., L-serine, L-alanine, D-glucose, D-mannose, and nickel chloride) are quite structurally dissimilar from clz, the Rluc substrate, suggesting that their inhibitory effects most likely result from some nonspecific mode of inhibition, rather than from direct competition with clz for access to the enzyme’s active site. For example, it is possible these molecules compromise protein fold, photonic processes, or form inhibitory aggregates that decrease the luminescent signal [33].

We considered the possibility that low concentrations of these molecules might not inhibit full-length Rluc, but still be potent enough to affect chemotactic signaling and elicit a meaningful response from the Rluc PCA. However, chemoeffector concentrations at the detection limit of the assay (∼1 µM) resulted in significant Rluc inhibition (data not shown), rendering the system untenable. Furthermore, the lack of a response to L-arginine (Figure 4A), a strong *V. cholerae* chemoattractant that should in principle decrease CheY3-RlucN/CheZ-RlucC interactions and the corresponding luminescent signal, suggests that the binding of these two fusion proteins may be irreversible. While this possibility is unexpected, there may be factors specific to this application (e.g., altered folding kinetics of Rluc fragments in bacteria, unforeseen effects of Rluc fusions on CheY3-CheZ phosphotransfer) that compromise the previously demonstrated reversibility of split luciferase complementation observed in eukaryotes.

Split luciferase is a powerful tool for measuring protein-protein interactions in live cells. However, our observations demonstrate an essential control for these studies: when investigating the effects of small molecules on dynamic protein-protein interactions, cells expressing the full-length luciferase must also be tested to ensure that compounds of interest truly inhibit protein interactions and not the activity of the reconstituted luciferase. A review of the split luciferase literature revealed that this control is seldom performed. Thus, we urge caution when applying this system to identify small molecule effectors of dynamic protein-protein interactions.

## Supporting Information

Figure S1
**Split Rluc signal is influenced by inducer concentration and Rluc substrate.** Luminescence generated by wild-type *V. cholerae* co-expressing CheY3-RlucN and CheZ-RlucC following induction with either 0.01% (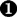) or 0.2% (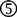) L-arabinose and treatment with native coelenterazine (clz) (*A*), or induction with 0.2% L-arabinose and treatment with either clz (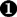) or benzyl-coelenterazine (clz-h, 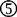) (*B*). Data are reported as a percentage of the highest initial signal intensity and represent the average of three technical replicates.(TIF)

Table S1 **Strains used in this study.**
(DOCX)

Table S2 **Plasmids used in this study.**
(DOCX)

Table S3 **Primers used in this study.**
(DOCX)
