# Supplementary figures and images for: Studies of Dynamic Protein-Protein Interactions in Bacteria Using Renilla Luciferase Complementation Are Undermined by Nonspecific Enzyme Inhibition
Source: PLoS One. 2012 Aug 15;7(8):e43175. doi: 10.1371/journal.pone.0043175 (PMC3419657; doi:10.1371/journal.pone.0043175)

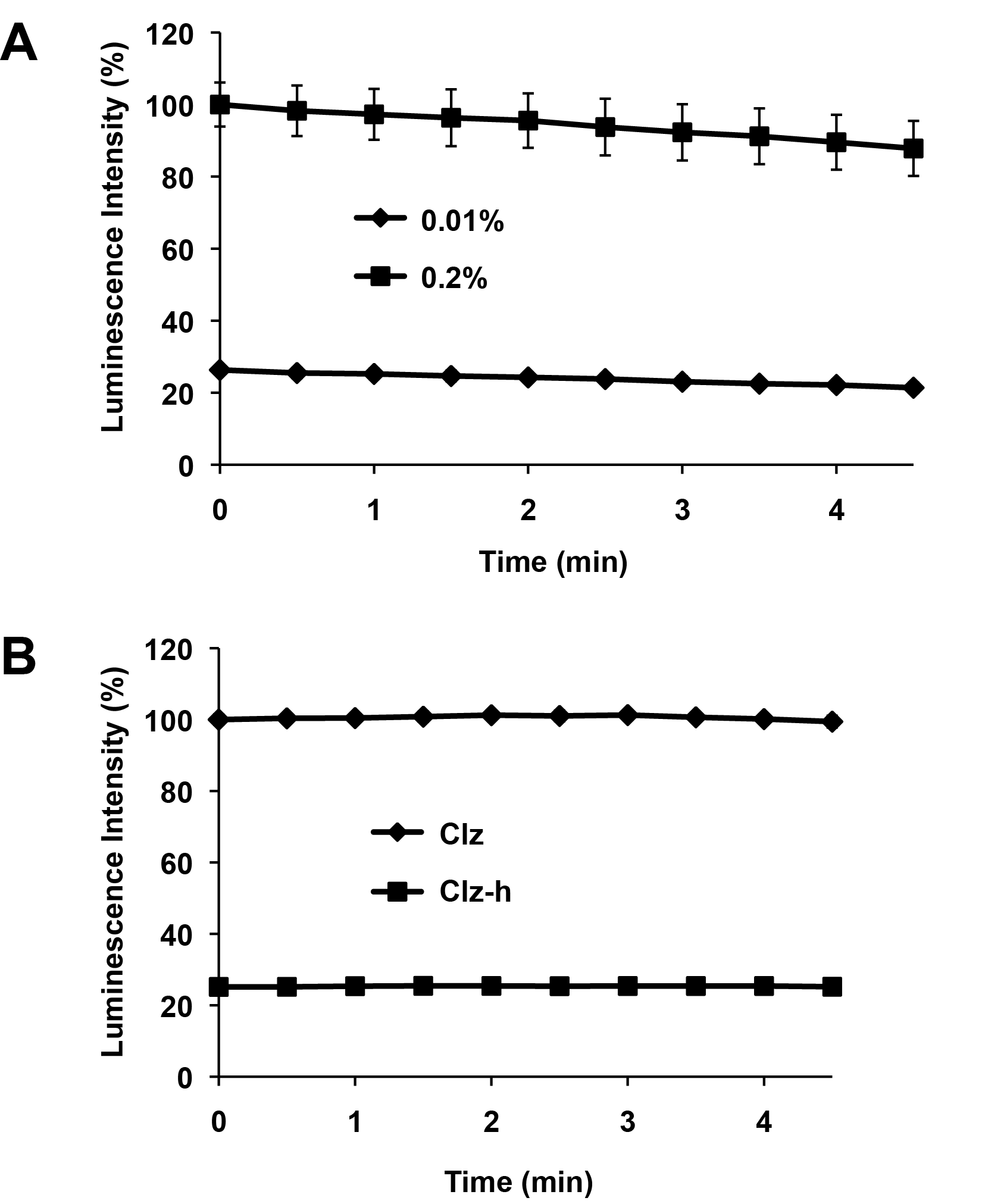

Supplement: Figure S1 — Split Rluc signal is influenced by inducer concentration and Rluc substrate. Luminescence generated by wild-type V. cholerae co-expressing CheY3-RlucN and CheZ-RlucC following induction with either 0.01% () or 0.2% () L-arabinose and treatment with native coelenterazine (clz) (A), or induction with 0.2% L-arabinose and treatment with either clz () or benzyl-coelenterazine (clz-h, ) (B). Data are reported as a percentage of the highest initial signal intensity and represent the average of three technical replicates. (TIF) [file pone.0043175.s001.tif]
