# Supplementary material for: Studies of Dynamic Protein-Protein Interactions in Bacteria Using Renilla Luciferase Complementation Are Undermined by Nonspecific Enzyme Inhibition
Source: PLoS One. 2012 Aug 15;7(8):e43175. doi: 10.1371/journal.pone.0043175 (PMC3419657; doi:10.1371/journal.pone.0043175)
Supplement: Table S1 — Strains used in this study. (DOCX) [file pone.0043175.s002.docx]

**Table S1. Strains used in this study.**

| **Strains** | **Relevant genotype/description** | **Reference** |
| --- | --- | --- |
| *Vibrio cholerae* C6706 | El Tor clinical isolate | [1] |
| *Vibrio cholerae* ∆*cheY3cheZ* | C6706 *lacZ^-^ ∆vc2064 ∆vc2065* | This work |
| *Vibrio cholerae* SR28 (∆*che2*) | C6706 *lacZ^-^* ∆*vc2059 ∆vc2060 ∆vc2061 ∆vc2062 ∆vc2063 ∆vc2064 ∆vc2065* | [2] |
| *Vibrio cholerae* SR33 (∆*che1che2che3*) | C6706 *lacZ^-^* *∆vc1394 ∆vc1395 ∆vc1396 ∆vc1397 ∆vc1398 ∆vc1399 ∆vc1400 ∆vc1401 ∆vc1402 ∆vc1403 ∆vc1404 ∆vc1405 ∆vc1406* ∆*vc2059 ∆vc2060 ∆vc2061 ∆vc2062 ∆vc2063 ∆vc2064 ∆vc2065 ∆vca1088 ∆vca1089 ∆vca1090 ∆vca1091 ∆vca1092 ∆vca1093 ∆vca1094 ∆vca1095 ∆vca1096* | This work |
| *Escherichia coli* DH5αλ*pir* | Cloning strain |  |
| *Escherichia coli* SM10λ*pir* | Conjugation donor | [3] |
| *Escherichia coli* MG1655 | *Escherichia coli* K12 |  |

**References**

1. Mandlik A, Livny J, Robins WP, Ritchie JM, Mekalanos JJ, et al. (2011) RNA-Seq-based monitoring of infection-linked changes in *Vibrio cholerae* gene expression. Cell Host Microbe 10: 165-174.

2. Ringgaard S, Schirner K, Davis BM, Waldor MK (2011) A family of ParA-like ATPases promotes cell pole maturation by facilitating polar localization of chemotaxis proteins. Genes Dev 25: 1544-1555.

3. Miller VL, Mekalanos JJ (1988) A novel suicide vector and its use in construction of insertion mutations: osmoregulation of outer membrane proteins and virulence determinants in *Vibrio cholerae* requires *toxR*. J Bacteriol 170: 2575-2583.
