# Supplementary material for: Studies of Dynamic Protein-Protein Interactions in Bacteria Using Renilla Luciferase Complementation Are Undermined by Nonspecific Enzyme Inhibition
Source: PLoS One. 2012 Aug 15;7(8):e43175. doi: 10.1371/journal.pone.0043175 (PMC3419657; doi:10.1371/journal.pone.0043175)
Supplement: Table S2 — Plasmids used in this study. (DOCX) [file pone.0043175.s003.docx]

**Table S2. Plasmids used in this study.**

| **Plasmid** | **Relevant genotype/description** | **Reference** |
| --- | --- | --- |
| pBAD33 | Cloning vector | [1] |
| pCVD442 | Allele exchange vector | [2] |
| pSR1020 | Allele exchange vector for deletion of *V. cholerae* chemotaxis cluster II genes | [3] |
| pSR1157 | Allele exchange vector for deletion of *V. cholerae* chemotaxis cluster I genes | This work |
| pSR1158 | Allele exchange vector for deletion of *V. cholerae* chemotaxis cluster III genes | This work |
| pCVD442-∆*cheY3cheZ* | Allele exchange vector for deletion of *V. cholerae* *cheY3* and *cheZ* genes | This work |
| pYNZC | pBAD33::*cheY3-rlucN*::*cheZ-rlucC* | This work |
| pRlucN-RlucC | pBAD33::*rlucN*::*rlucC* | This work |
| pRluc | pBAD33::*rluc* | This work |

**References**

1. Guzman LM, Belin D, Carson MJ, Beckwith J (1995) Tight regulation, modulation, and high-level expression by vectors containing the arabinose P_BAD_ promoter. J Bacteriol 177: 4121-4130.

2. Donnenberg MS, Kaper JB (1991) Construction of an *eae* deletion mutant of enteropathogenic *Escherichia coli* by using a positive-selection suicide vector. Infect Immun 59: 4310-4317.

3. Ringgaard S, Schirner K, Davis BM, Waldor MK (2011) A family of ParA-like ATPases promotes cell pole maturation by facilitating polar localization of chemotaxis proteins. Genes Dev 25: 1544-1555.
