# Supplementary material for: Studies of Dynamic Protein-Protein Interactions in Bacteria Using Renilla Luciferase Complementation Are Undermined by Nonspecific Enzyme Inhibition
Source: PLoS One. 2012 Aug 15;7(8):e43175. doi: 10.1371/journal.pone.0043175 (PMC3419657; doi:10.1371/journal.pone.0043175)
Supplement: Table S3 — Primers used in this study. (DOCX) [file pone.0043175.s004.docx]

**Table S3. Primers used in this study.**

| **Primer** | **Sequence (5'🡪3')***^a^* | **Description** |
| --- | --- | --- |
| SKH-51 | TTCA**TCTAGA**ATACCGAGAACACCAAGTGGACATTTGGA | Reverse primer for region downstream of *cheY3*; XbaI site |
| SKH-52 | TTCATGTTTTTATTCAAAATTGCCTCCACTGAGT | Forward primer for region downstream of *cheY3* |
| SKH-53 | GAATAAAAACATGAATTTGGCGCAGCGCAAAAAGAACA | Reverse primer for region upstream of *cheZ* |
| SKH-54 | TACC**TCTAGA**GATATCACCGTTGAGATCAGAATTGGTTACTG | Forward primer for region upstream of *cheZ*; XbaI site |
| SKH-61 | TACAC**TCTAGA**TAAGGAGGATTTATAAATGATTTCATTAGAACAGGCTAAAGAACTCGT | Forward primer for *cheZ*; XbaI site and RBS |
| SKH-67 | TACAC**GGTACC**GTGGAGGCAATTTTGAATAAAAACATGAAGATCC | Forward primer for *cheY3*; KpnI site |
| SKH-68 | TCCGGAGGACCCACCACCTCCAGAGCCACCGCCACCATCGATTAAACGCTCAAAAATTTTGTCTAATTTTTCTTTAAGC | Reverse primer for *cheY3* with linker |
| SKH-69 | ATCGATGGTGGCGGTGGCTCTGGAGGTGGTGGGTCCTCCGGAGCTTCGAAAGTTTATGATCCAGAACAAAGG | Forward primer for *rlucN* with linker |
| SKH-70 | CATAG**TCTAGA**TTATAAATTAAGAAGTTCAAACCATGCAGTAAGATATTTGT | Reverse primer for *rlucN*; XbaI site |
| SKH-71 | TACAC**GGTACC**ATGGCTTCGAAAGTTTATGATCCAGAACAAA | Forward primer for *rlucN*; KpnI site |
| SKH-74 | TCCGGAGGACCCACCACCTCCAGAGCCACCGCCACCATCGATAAATCCAAGACTCGATAACAGATCGTCAAC | Reverse primer for *cheZ* with linker |
| SKH-75 | ATCGATGGTGGCGGTGGCTCTGGAGGTGGTGGGTCCTCCGGACCAAAGAAGATCATTTTTGTCGGCCATGA | Forward primer for *rlucC* with linker |
| SKH-76 | TACAC**GCATGC**TTATTGTTCATTTTTGAGAACTCGCTCAACG | Reverse primer for *rlucC*; SphI site |
| SKH-83 | CACAG**TCTAGA**TAAGGAGGATTTATAAATGCCAAAGAAGATCATTTTTGTCGGCCATGA | Forward primer for *rlucC* with 5' ATG; XbaI site and RBS |
| che1-aa | CCCC**TCTAGA**AAACGCTTCCGCATGGATGCC | Reverse primer for region downstream of *vc1406*; XbaI site |
| che1-bb | AAAAAAGCGGCCGCAAAAAATTCAGCATGCAGCGGAAAATGTAC | Forward primer for region downstream of *vc1406* |
| che1-cc | TTTTTTGCGGCCGCTTTTTTAAAGCAATCGACGTTATCCACTTC | Reverse primer for region upstream of *vc1394* |
| che1-dd | CCCC**TCTAGA**TGACTTATTTGGCGCAACTGTTCC | Forward primer for region upstream of *vc1394*; XbaI site |
| che3-aa | CCCC**TCTAGA**AGGGTGTTCGTTATTGAGTTTGATC | Reverse primer for region downstream of *vca1096*; XbaI site |
| che3-bb | AAAAAAGCGGCCGCAAAAAAACTTTTGCCATAGTGTTTCCCTCA | Forward primer for region downstream of *vca1096* |
| che3-cc | TTTTTTGCGGCCGCTTTTTTTGGTGTCCAGCAACTGAATACC | Reverse primer for region upstream of *vca1088* |
| che3-dd | CCCC**TCTAGA**TGGCAATGAGTACGCTACGCTC | Forward primer for region upstream of *vca1088*; XbaI site |
| *^a^*Enzyme restriction sites appear in boldface text. Ribosome-binding sites (RBS) are underlined. | | |
